# Editorial: Recent Advances in the Evolution of Euarchontoglires

**DOI:** 10.3389/fgene.2021.773789

**Published:** 2021-10-11

**Authors:** Łucja Fostowicz-Frelik, Deyan Ge, Irina Ruf

**Affiliations:** ^1^ Department of Evolutionary Paleobiology, Institute of Paleobiology, Polish Academy of Sciences, Warsaw, Poland; ^2^ Key Laboratory of Vertebrate Evolution and Human Origins, Institute of Vertebrate Paleontology and Paleoanthropology, Chinese Academy of Sciences, Beijing, China; ^3^ Center for Excellence in Life and Paleoenvironment, Chinese Academy of Sciences, Beijing, China; ^4^ Key Laboratory of Zoological Systematics and Evolution, Institute of Zoology, Chinese Academy of Sciences, Beijing, China; ^5^ Abteilung Messelforschung und Mammalogie, Senckenberg Forschungsinstitut und Naturmuseum Frankfurt, Frankfurt am Main, Germany

**Keywords:** lagomorphs, primates, rodents, behavior, evolution, genetics, morphology

## Introduction

Euarchontoglires, recognized two decades ago in molecular studies (Murphy et al., 2001), is the most numerous and arguably, one of most important clades of placental mammals. First, Euarchontoglires include extremely variable and numerous rodents, the most speciose extant mammalian clade on Earth. Rodent diversity and array of adaptations found in this clade allow us to study genetics and diversification, mechanisms and the evolutionary patterns of speciation. Second, Euarchontoglires include primates (among them humans), which are characterized by notably increased encephalization. This major adaptive transformation alone warrants our attention. Third, the considerable antiquity of Euarchontoglires with over 63 million years of evolutionary history documented in the fossil record also makes this clade important for understanding mammalian evolution.

Apart from rodents (∼2,500 living species) and primates (∼300 species), representing approximately half of extant mammalian species nowadays (Wilson et al., 2016) and supplying us with excellent models to study extremely successful mammalian radiations, Euarchontoglires include also a few less numerous groups such as lagomorphs (rabbits, hares, and pikas), scandentians (tree-shrews), and dermopterans, otherwise known as colugos or flying lemurs. These understudied clades certainly contribute to our understanding of the Euarchontoglires evolution and early divergence of major lineages.

That said, the exceptional diversity of both fossil and living taxa of Euarchontoglires can be seen as a challenge, because the multitude of forms and adaptations found in these placentals makes their origins and evolution sometimes hard to decipher. Particularly characteristic of the Glires clade (lagomorphs + rodents) is their frequently convergent morphological evolution, which we now start testing quantitatively (e.g., Morris et al., 2018).

For this Research Topic, we collected 14 original research papers and reviews concerning evolution of Euarchontoglires at molecular, morphological, and behavioral levels, and conservation issues in this clade.

## Euarchontoglires in General

Two papers in this volume address the broader context of Euarchontoglires evolution. Among several important issues which remain poorly studied, unresolved or debatable for this clade are convergence, reconstruction of ancestral morphotype and comparative genome-wide studies. Geng et al. investigate phenotypic convergence of locomotor modes across Euarchontoglires (122 species excluding Lagomorpha) on the basis of some forelimb skeleton indices. Song et al. present a survey of microsatellite DNA composition and its diversity within all major clades of Euarchontoglires including Scandentia and Dermoptera.

## Primates

Since the last two decades it has become evident that evolutionary biology and conservation issues are greatly interconnected. Thus, a sizeable portion of this volume is dedicated to primate research at the interface of molecular research and species at-risk. Guo et al. investigated the genetic structure of critically endangered Hainan gibbon (*Nomascus hainanus*), the rarest primate in the world. Endangered or critically-endangered snub-nosed monkeys (*Rhinopithecus*) were the subject of three studies. Zhang et al. investigate the role of major histocompatibility complex (MHC) genes in mate choice in the golden snub-nosed monkey (*R*. *roxellana*). Li et al. tackle the genetic structure of the Qinling Mountains population of the same species. Kuang et al. present a comprehensive study concerning the genomic impact of population dwindling of these monkeys across the entire genus, and find deleterious mutations that are affecting immunity especially in smaller populations. Finally, Yousaf et al. in their mini-review share the current state of the art in the field of great apes genome analysis, showing variety of approaches, recent views at the species relationships and the evolutionary history of the closest human relatives from the standpoint of molecular studies, as well as the emergence of modern human genome.

Behavior has been frequently studied in primates, due to size and structure of their brain resulting in social organization. Xia et al. investigate social status in female Tibetan macaques (*Macaca thibetana*) as expressed by grooming relationships within the group.

## Rodentia

Wölfer et al. investigate locomotion in Swinhoe’s striped squirrel (*Tamiops swinhoei*). They hypothesize that asymmetrical gait may have been characteristic of earliest representatives of Euarchontoglires.

Traditionally, the fossil record has been established as a tool for understanding macroevolutionary changes. Due to very dense sampling, Kimura et al. in their paleobiological study of murine rodents were able to document morphological evolution in a rodent species with a resolution of hundreds of thousands of years.

A complex social organization is also characteristic of some rodents. Caspar et al. explore how and to what degree sex and breeding status influence skull characters in a eusocial mole-rat (*Fukomys*). Their study contributes to a deeper understanding of morphofunction and sexual dimorphism in Bathyergidae.

## Lagomorpha

Although usually overshadowed by rodents, their sister clade, lagomorphs are important for our understanding of non-primate Euarchontoglires evolution. Two papers focus on the cranial anatomy of one of the most common and widespread North American Paleogene lagomorph *Palaeolagus*. This early “rabbit” waited, perhaps too long, for a new appraisal since Wood’s (1940) classic study. Using modern digital imaging techniques, Wolniewicz and Fostowicz-Frelik present the general anatomy of the *Palaeolagus* skull, complemented by Ruf et al. on the nasal and auditory regions in detail. In their review, Kraatz et al. describe lagomorphs as a good model system to study multiple evolutionary patterns of morphological change. This truly collaborative effort shows not only how much has been learned on lagomorphs recently, but may suggest new prospects for future research.

## Perspectives

The above contributions give us only a snapshot of recent developments in Euarchontoglires studies. Here we offer a handful of predictions as to future research directions in the field (Although we refrain from mentioning in detail recent developments in molecular biology). First, it is evident from anatomical studies on the skeleton that it is important to investigate more closely soft tissues (muscles, blood vessels and brain in particular). Second, methods should be used not only to generate image data but also to simulate and model function of various systems. Even established methods, e.g., finite element analyses (FEA) have been employed only rarely to understand the biomechanics in non-primate Euarchontoglires. Third, there is a whole evo-devo modern approach that links ontogeny with morphological change. Moreover, new sequencing methods have resulted in generation of large genomic datasets, which not only helps reconstruct more robust phylogenies of Euarchontoglires, but also facilitates studies on adaptive evolution and demographic histories, and the mechanisms underlying speciation and key traits evolution.

However, there is a lack of balance in the coverage of existing diversity of Euarchontoglires; the primates (especially) and rodents are quite well explored, whereas other clades, such as lagomorphs, scandentians and dermopterans are rather neglected. Thus, one of immediate goals should be to focus on the understudied groups of Euarchontoglires in order to bring research on these clades to the standards now available only for primates.

Last but not least, we are very grateful to all the authors and reviewers who generously contributed their work and time to this volume. Hopefully, our collection will stir further interest in these fascinating placental mammals and will provide a starting point for new research or even revisiting old questions.
